# Formation mechanism of guided resonances and bound states in the continuum in photonic crystal slabs

**DOI:** 10.1038/srep31908

**Published:** 2016-08-25

**Authors:** Xingwei Gao, Chia Wei Hsu, Bo Zhen, Xiao Lin, John D. Joannopoulos, Marin Soljačić, Hongsheng Chen

**Affiliations:** 1 The Innovative Institute of Electromagnetic Information and Electronic, Zhejiang University, Hangzhou 310027, China; 2 The Electromagnetics Academy at Zhejiang University, Zhejiang University, Hangzhou 310027, China; 3 Research Laboratory of Electronics, Massachusetts Institute of Technology, Cambridge, Massachusetts 02139, USA; 4 Department of Applied Physics, Yale University, New Haven, Connecticut 06520, USA; 5 Physics Department and Solid State Institute, Technion, Haifa 32000, Israel

## Abstract

We develop a formalism, based on the mode expansion method, to describe the guided resonances and bound states in the continuum (BICs) in photonic crystal slabs with one-dimensional periodicity. This approach provides analytic insights to the formation mechanisms of these states: the guided resonances arise from the transverse Fabry–Pérot condition, and the divergence of the resonance lifetimes at the BICs is explained by a destructive interference of radiation from different propagating components inside the slab. We show BICs at the center and on the edge of the Brillouin zone protected by symmetry, BICs at generic wave vectors not protected by symmetry, and the annihilation of BICs at low-symmetry wave vectors.

Conventionally, the confinement of waves is achieved by spectrally separating the bound state away from the continuum of radiating waves that can carry energy away—examples include electronic bound states at negative energies and light guided below the light line or inside a photonic bandgap. Bound states in the continuum (BICs) are special states that remain localized and have infinite lifetimes even though they reside inside the continuum^1^. Historically, BIC was first proposed by von Neumann and Wigner for an electron in an engineered potential^2^, although such an electron BIC has never been achieved. More recently, optical BICs have been experimentally realized in a range of photonic systems^3^,^4^,^5^,^6^,^7^,^8^,^9^. In periodic structures, BICs may be found by varying the incident angle without tuning the structure, which makes their realization relatively simple^3^,^5^,^6^,^10^,^11^,^12^,^13^,^14^,^15^,^16^,^17^,^18^,^19^,^20^,^21^,^22^ (Note: Originally, BICs are proposed as localized states integrable in all the three dimensions^2^. But in periodic structure, BICs are extended in periodical directions while spatially confined in other directions). Photonic crystal (PhC) slabs—dielectric slabs with periodically modulated refractive index^10^,^17^,^23^,^24^—are particularly attractive given their macroscopic sizes and ease of fabrication. The guided resonances and BICs in PhC slabs have been used for a wide range of applications such as lasers^25^,^26^,^27^,^28^,^29^, sensors^12^,^30^,^31^, and filters^32^. When BICs do exist, their robustness can be explained by the zero-crossing of radiating amplitudes^6^ or, more generally, by their topological charges^18^. However, this does not reveal the physical mechanism that suppresses radiation and leads to the localization. In the presence of multiple resonances, the disappearance of radiation can be explained by the destructive interference of the radiating waves from different resonances^7^,^12^,^13^,^14^,^15^,^33^,^34^. When only one resonance is present, an explanation based on destructive interference should still be possible, but it is no longer clear which sets of waves are interfering. Furthermore, the theoretical studies of BICs have largely relied on numerical simulations that are time consuming and provide little insight.

Here, we develop a mode expansion method that explains the formation mechanism of guided resonances and BICs in PhC slabs. In this formalism, guided resonances require the round-trip transverse phase shift for each in-slab propagating mode to be an integer multiple of 2π, and BICs arise from the destructive interference of radiation from different propagating waves inside the slab. This method is also capable of calculating the resonance frequency, quality factor, and field profile efficiently with no approximation other than a truncation of the basis size, and we validate these results with finite-difference time-domain (FDTD) simulations. For PhC slabs in a periodic inhomogeneous backgrounds, we find symmetry-protected BICs both at the center and on the edge of the Brillouin zone, in addition to those inside the Brillouin zone where they are not protected by symmetry. Although we only show examples for systems with one dimensional periodicity, our mode expansion method can be extended to systems that are periodic in two dimensions.

## Methods

**Figure 1:**
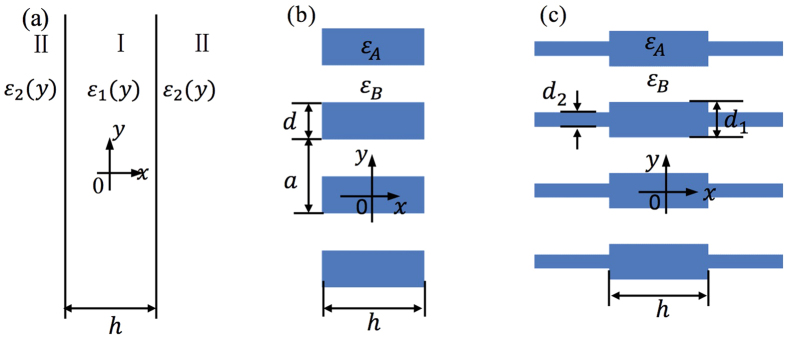
(**a**) Structure: a 1D-periodic photonic crystal (PhC) slab with permittivity *ε*_1_(*y*) surrounded by a dielectric media with permittivity *ε*_2_(*y*). *ε*_1_ and *ε*_2_ have the same period of *a*, *ε*_1_(*y* + *a*) = *ε*_1_(*y*), *ε*_2_(*y* + *a*) = *ε*_2_(*y*). (**b**) Schematic of a 1D PhC slab embedded in homogeneous dielectric medium. (**c**) Schematic of a 1D PhC slab embedded in a periodic dielectric background.

For simplicity, we consider TM modes (*H*_*x*_, *H*_*y*_, *E*_*z*_) in a PhC slab that is periodic in *y* and uniform in *z* (Fig. 1a); the generalization to TE modes (*E*_*x*_, *E*_*y*_, *H*_*z*_) and to PhC slabs with two-dimensional periodicity is straightforward. We consider structures that are mirror-symmetric in the normal direction *x* (this symmetry is necessary for reducing the number of radiation channels^6^) and where the slab permittivity *ε*_1_(*y*) is uniform in *x* (common in most fabricated structures of PhC slabs). Here we consider two cases: the permittivity of the surrounding medium *ε*_2_(*y*) is a constant (Fig. 1b) or is periodic with the same period *a* as the slab (Fig. 1c). Inside (|*x*| < 0.5*h*) and outside (|*x*| > 0.5*h*) the slab, the structure is uniform in *x*, so the fields can be expanded in the eigenmodes of *ε*_1_(*y*) and *ε*_2_(*y*) with a sinusoidal dependence along *x*; the expansion coefficients can be determined by continuity at the slab surface |*x*| = 0.5*h*. Specifically, with an outgoing boundary condition in *x*, an even-in-*x* TM mode with wave vector *k*_*y*_ can be written as^35^


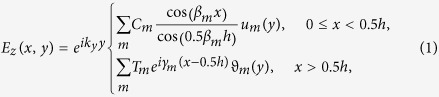


where *h* is the slab thickness, the cosine inside the slab guarantees the even-in-*x* symmetry, and the complex exponential outside the slab guarantees the outgoing boundary condition. TM modes satisfy the wave equation 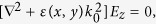 where *k*_0_ = *ω*/*c*, *ω* is the frequency and *c* is the vacuum speed of light; inserting Eq. (1) into this wave equation, we find that the eigenfunctions and propagation constants *u*_*m*_(*y*), *ϑ*_*m*_(*y*), *β*_*m*_and *γ*_*m*_ inside and outside the slab satisfy


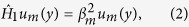



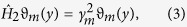


where 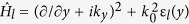 for *l* = 1, 2 are the Hermitian operators governing the wave equation for the layers inside and outside the slab, subject to periodic boundary condition *u*_*m*_(*y* + *a*) = *u*_*m*_(*y*), *ϑ*_*m*_(*y* + *a*) = *ϑ*_*m*_(*y*). For a given frequency and *k*_*y*_, there will be a finite number of eigenmodes with 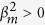 (or 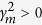) that propagate in the *x* direction, with an infinite number of eigenmodes with 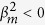 (or 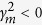) that are evanescent in *x*. Similar expansion methods were used previously for water waves^21^ and for quantum waveguides^36^. Odd-in-*x* TM modes can be written similarly by replacing the cosine in Eq. (1) with sine, and TE modes can be treated by replacing 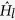 with that for *H*_*z*_. *C*_*m*_ and *T*_*m*_ are coefficients of the eigenmode expansion, and they can be determined via the continuity of *E*_*z*_ and ∂*E*_*z*_/∂*x* at *x* = 0.5*h*, which requires


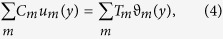



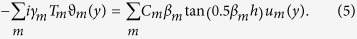


The standing waves inside the slab [the cos (*β*_*m*_*x*) in Eq. (1)] are superposition of waves propagating in +*x* and in –*x* directions, so one can interpret the in-slab fields as waves circulating within the slab with reflection and transmission at the two slab surfaces. The transverse phase shift for every propagating component is an integer multiple of 2π after a round trip with two reflections, which is the same resonance condition as the Fabry–Pérot resonances in uniform dielectric slabs; the difference is that here multiple propagating components are coupled due to the periodicity in *y*.

While the two sets of eigenmodes {*ϑ*_*m*_} and {*u*_*m*_} each form an infinite-dimensional basis, the high-order ones correspond to fast oscillating fields that are negligible at low frequencies. Therefore, in our calculations, we truncate down to *M* terms by expanding the eigenmodes in an *M*-dimensional basis (details below). In the truncated basis, the transformation between the two bases {*ϑ*_*m*_} and {*u*_*m*_} is given by to an *M* × *M* matrix **P** such that 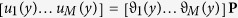. In this way, the continuity condition Eqs (4, 5) can be written in matrix form as


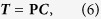



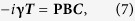


where ***T*** = [*T*_1_, …, *T*_*M*−1_, *T*_*M*_]^T^, ***C*** = [*C*_1_, …, *C*_*M*−1_, *C*_*M*_]^T^, and **γ**, **B** are diagonal matrices **γ** = Diag(*γ*_1_, …, *γ*_*M*_), **B** = Diag (*β*_1 _tan (0.5*β*_1_*h*), …, *β*_*M*_ tan (0.5*β*_*M*_*h*)). Note that the matrix **B** is purely real since 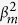 is real for all *m*. Eq. (4) is a linear equation group for vectors ***T*** and ***C***. Substituting Eq. (6) into Eq. (7) yields (*i***γP** + **PB**)***C*** = **0**. Non-trivial solutions exist when


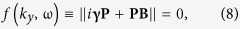


where ||*|| denotes the determinant of the matrix. Therefore, solving for Eq. (8) for a given *k*_*y*_ yields the dispersion relation *ω* (*k*_*y*_) for arbitrary resonances and BICs, as well as regular bound states. The vector ***C*** corresponding to the zero determinant and the associated vector ***T*** from Eq. (6) yield the field profile as given in Eq. (1). At frequencies in the continuum spectrum of the surrounding medium (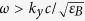 for a homogeneous medium), some of the *γ*_*m*_’s are real, and *f (k*_*y*_, ω) is generally complex-valued; in such region finding the zeroes of *f (k*_*y*_, ω) requires searching for solutions on the lower half of the complex-frequency plane with *ω*_*r*_ = Re(*ω*) and *ω*_*i*_ = Im(*ω*) being the parameters. The imaginary part of the frequency is the decay rate, and the quality factor of the resonance is *Q* = −*ω*_*r*_/(2*ω*_*i*_).

The transformation matrix **P** is deduced from Eqs (2, 3). Inspired by ref. 24, we expand both sides of Eqs (2, 3) in Fourier series and truncate to Fourier orders from –*N* to *N* (with a total of *M* = 2*N* + 1 terms). Then, Eqs (2, 3) can be written as matrix equations


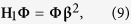



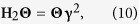


with **β** = Diag(*β*_1_, …, *β*_*M*_), and **H**_*l*_ is an *M* × *M* matrix whose (*m*, *m*′)-th element is 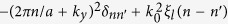, where n = *m* − *N* − 1, n′ = *m*′ − *N* − 1, and *ξ*_*l*_ (*n*) is the *n*-th Fourier coefficient of *ε*_*l*_ (*y*). The *m*-th column of **Φ** (or **Θ**) contains the –*N*-to-*N* Fourier coefficients of *u*_*m*_ (or *ϑ*_*m*_). **Φ** and **Θ** are transform matrices connecting {*u*_*m*_} and {*ϑ*_*m*_} to the same basis with plane-wave elements, hence ***P*** = **Θ**^−1^**Φ**. Note that when the permittivity is real and mirror symmetric in *y*, 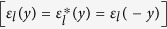, the matrices **H**_*l*_ are real symmetric, so **β**^2^ and **γ**^2^ are real; moreover, **Φ**, **Θ**, and **P** can be chosen to be purely real.

BICs arise when the decay rate γ = −2ω_*i*_ of a resonance becomes zero, or equivalently when the amplitudes of the radiating waves vanish: *T*_*m*′_ = 0 for all the *m*′ with 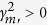. From Eq. (6), *T*_*m*′_ is given by


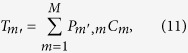


where 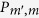 is the (*m*′,*m*)’s element of matrix **P**, which is the *m*′-th component of the in-slab eigenmode *u*_*m*_ in the basis of {*ϑ*_*m*′_}. Equation (11) reveals that the radiating wave of port *m*′ comes from interference of all the contributions from {*u*_*m*_} to the radiating mode *ϑ*_*m*′_. Therefore, *T*_*m*′_ = 0 is a result of destructive interference from the in-slab eigenmodes to the radiation modes.

Numerically it can be ambiguous to determine whether ω_*i*_ is very small or identically zero. Therefore, we use a slightly modified scheme to look for exact BICs. Define *f* ′ as 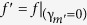: the propagation constants of the radiating waves *γ*_*m*′_ are artificially set to zero; this function *f* ′ is purely real for a lossless dielectric structure that is symmetric in *y* (where **H**_*l*_ is real symmetric). A BIC not only satisfies *f* = 0; it also satisfies *f* ′ = 0 since *T*_*m*′_ = 0 for a BIC, and from Eq. (5) it can be seen that setting *γ*_*m*′_ to zero does not change the solution. Therefore, to search for BICs, we first solve the real-valued equation *f* ′(*k*_*y*_, ω) = 0 at each *k*_*y*_ for a real-valued frequency ω; the solution also provides a mode profile given by ***T*** and ***C***. However, such a mode profile will only satisfy the continuity condition, Eqs (4 and 5), if *T*_*m*′_ = 0. In this work, we study the frequency range where there is only one leaky channel (only one *m*′ with 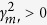), and we perform a root finding with *k*_*y*_ being the free parameter to look for solutions of *f* ′ = 0 where the amplitude of this radiation channel vanishes, *T*_*m*′_ = 0. Once found, such a solution will be a true bound state at a purely real frequency and with no radiation.

## Results

### Photonic crystal slab in a homogeneous background

**Figure 2:**
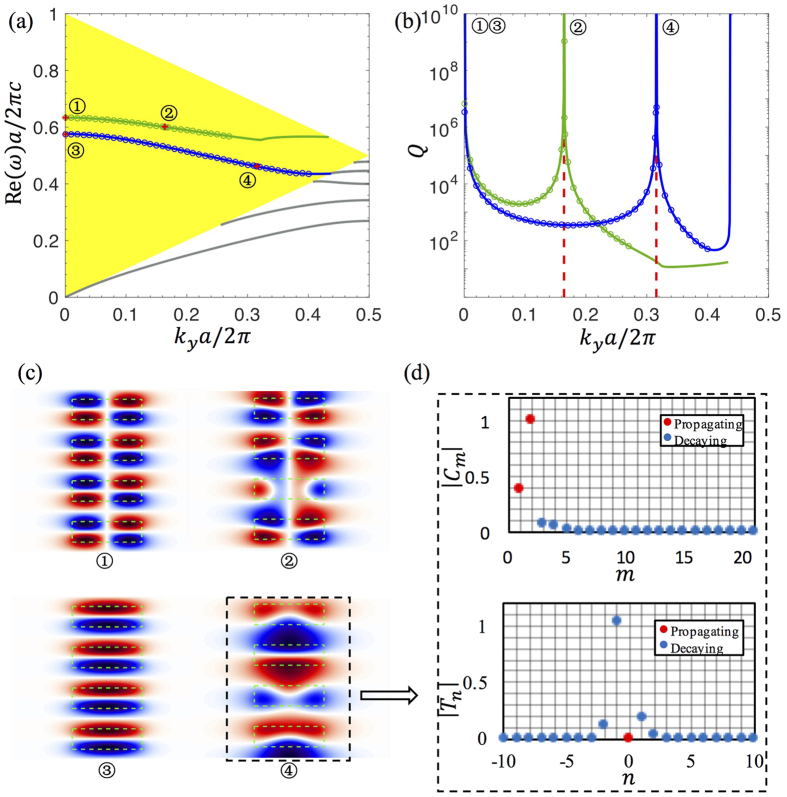
(**a**) Band structure and (**b**) quality factors of the guided resonances and BICs in the system illustrated in Fig. 1b. (**a**) Yellow shaded area is where there is only one leaky channel in the surrounding medium. The blue (green) solid curve is the dispersion of guided resonances that are even (odd) in *x*. BICs are marked with red plus signs: ① and ③ are protected by symmetry, while ② and ④ are not. Circles are FDTD simulation results. The grey curves are guided modes below the light line. Red dashed lines in (**b**) mark the location of BICs not protected by symmetry. (**c**) Electric field patterns of the BICs. (**d**) Magnitudes of the mode-expansion coefficients in the slab ***C*** (upper panel) and outside the slab ***T*** (lower panel) for the BIC ④. Red dots are components propagating in the *x* direction (where *β*_*m*_ or *γ*_*m*_ is real); blue dots are evanescent components with imaginary wave vector along the *x* direction. The structural parameters are: *ε*_*A*_ = 4.9, *ε*_*B*_ = 1, *h* = 1.4*a*, *d* = 0.5*a*.

In this work we study two systems. The first one is a layered slab in a homogeneous medium (Fig. 1b). It consists of a sequence of dielectric rectangles of size *d* × *h* with permittivity *ε*_*A*_, surrounded by a homogeneous material with permittivity *ε*_*B*_. The eigenmodes in the homogeneous medium are simply plane waves with 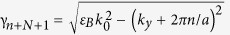 and **Θ** being an identity matrix. In Fig. 2a, the region with one leaky channel (one real *γ*_*m*′_) is shaded in yellow. In the slab, the Fourier coefficients of the permittivity is *ξ*_1_(*n*) = (*d*/*a*)(*ε*_*A*_ − *ε*_*B*_) sin c (*nd*/*a*) + *ε*_*B*_*δ*_*n*_. For the basis truncation, we take *M* = 21 Fourier terms and eigenmodes (*N* = 10), which is enough for the results to converge within the frequency range we consider. As an example, we take *ε*_*A*_ = 4.9 (this is a reasonably small dielectric constant and is close to many common optical materials such as silicon nitride, zinc oxide, gallium nitride, indium tin oxide, and diamond), *ε*_*B*_ = 1, *d* = 0.5*a*, and *h* = 1.4*a*. By solving Eq. (8), we obtain the band structure Re(*ω*) and the quality factor *Q* = −*ω*_*r*_/(2*ω*_*i*_) of the resonances, shown as solid curves in Fig. 2a,b. As a validation, we also perform finite-difference time-domain (FDTD) simulations for the same structure. The FDTD simulations are carried out at a spatial resolution of 32^2^ points per area of *a*^2^ (high enough for the calculated *Q* to converge) and take significantly longer than our method; the results, shown as circles in Fig. 2a,b, are in perfect agreement with our method.

The quality factor diverges at the BICs. Non-symmetry-protected BICs occur at *k*_*y*_ = 00.3156 (2*π*/*a*) for even-in-*x* modes (blue curve) and at *k*_*y*_ = 0.1640 (2*π*/*a*) for odd-in-*x* modes (green curve). Symmetry protected BICs can be found at the Γ point (*k*_*y*_ = 0), where radiation vanishes because *E*_*z*_ is odd in *y* for the resonance but even in *y* for the radiating wave. Such symmetry-incompatibility mechanism also holds at the edge of the Brillouin zone (*k*_*y*_ = *π*/*a*), but at the zone edge of this system modes are either regular bound states (below the yellow shaded area) or states with multiple leaky channels (above the yellow shaded area) for which BICs are harder to come by. We will show zone-edge BICs in the second system. Fig. 2c shows the field profiles of the four BICs.

In Fig. 2d, we plot the coefficients ***C*** and ***T*** of the even-in-*x* non-symmetry-protected BIC. Inside the slab (upper panel), the amplitudes ***C*** are dominated by two propagating modes (shown in red). Outside the slab (lower panel), there is only one propagating mode, and its amplitude vanishes at the BIC. The amplitude of the propagating mode outside the slab is the transmission from the in-slab modes, as shown in Eq. (11). Therefore, the disappearance of radiation arises from destructive interference of the transmission from the in-slab modes, which, as shown in the upper panel, primarily consist of two propagating modes.

### Photonic crystal slab in a periodically-modulated background

**Figure 3:**
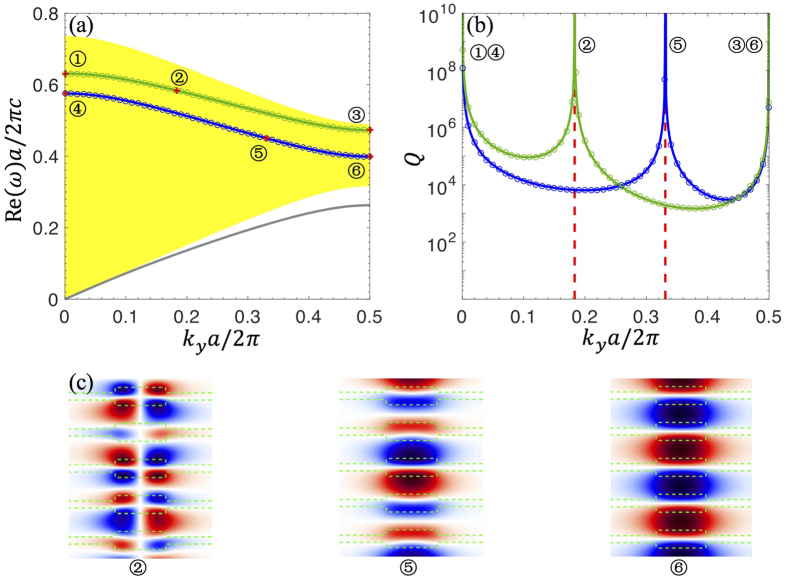
(**a**) Band structure and (**b**) quality factors of the guided resonances in the system illustrated in Fig. 1c. The convention is the same as Fig. 2. BICs are labeled by numbers; among them ①, ③, ④ and ⑥ are protected by symmetry, while ② and ⑤ are not. (**c**) Electric field patterns of the BICs labeled by ②, ⑤, ⑥. The structural parameters are: *ε*_*A*_ = 4.9, *ε*_*B*_ = 1, *h* = 1.4*a*, *d*_1_ = 0.5*a*, *d*_2_ = 0.2*a*.

The second system we consider is a PhC slab surrounded by a periodically-modulated background (Fig. 1c). The out-of-slab region has the same period as the slab but with a different filling fraction. We consider *ε*_*A*_ = 4.9, *ε*_*B*_ = 1, *d*_1_ = 0.5*a*, *d*_2_ = 0.2*a*, *h* = 1.4*a*. Figure 3a shows the band structure obtained from Eq. (8), with yellow shading over the region with one leaky channel (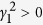, 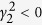); the corresponding quality factor is shown in Fig. 3b. Results from FDTD simulations are shown as circles in Fig. 3a,b; again the simulation results quantitatively agree with our method.

In this structure, symmetry-protected BICs can be found both at the Γ point (*k*_*y*_ = 0) and at the zone edge (*k*_*y*_ = *π*/*a*) inside the yellow-shaded region. The zone-edge BICs are possible because the periodic modulation in the background breaks the degeneracy of the propagating waves on the zone edge and opens up a finite yellow-shaded region where only one leaky channel is present. On the zone edge and within this region, the leaky wave is even under mirror flip around *y* = 0, but the BICs are odd (as can be seen from the mode profile in Fig. 3c) so they decouple from radiation. Meanwhile, non-symmetry-protected BICs still exist, as marked by red dashed lines in Fig. 3b and with the mode profiles shown in Fig. 3c.

**Figure 4:**
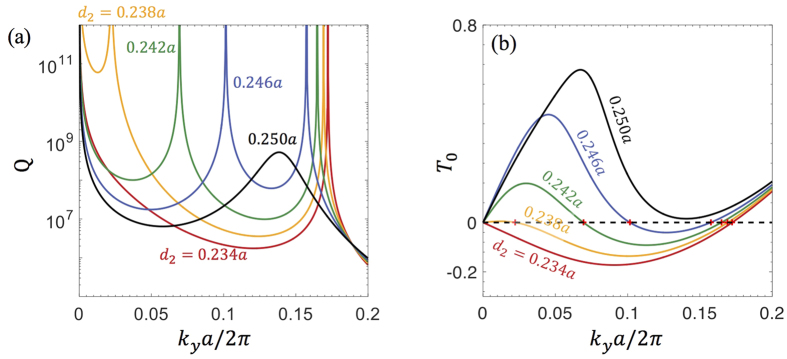
Annihilation of BICs. (**a**) Quality factors and (**b**) radiation coefficient *T*_0_ of the odd-in-*x* band for varying filling fractions *d*_2_/*a* in the background. In (**b**), BICs not protected by symmetry are labeled by red plus signs, which correspond to the infinite-*Q* peaks in (**a**). Two such BICs annihilate each other near *k*_*y*_ = 0.14 (2*π*/*a*) as *d*_2_/*a* increases past 0.25. The structural parameters are the same as Fig. 3 except for *d*_2_.

In this system, we can observe an interesting phenomenon that two BICs annihilate at low-symmetry *k* points. On the odd-in-*x* band, as we vary the filling fraction in the background from *d*_2_/*a* = 0.23 to *d*_2_/*a* = 0.25, we observe that another non-symmetry-protected BIC emerges from the Γ point, moves along the *k*_*y*_ axis, and then annihilates with the other non-symmetry-protected BIC near *k*_*y*_ = 0.14 (2*π*/*a*). The annihilation removes both BICs and leaves behind a finite peak in *Q*, as shown in Fig. 4a. The annihilation can be understood from the radiation coefficient *T*_0_, which we plot in Fig. 4b. Each zero-crossing of *T*_0_ corresponds to a BIC. Since *T*_0_ is expected to change continuously, two adjacent zero-crossings must have opposite slope and will cancel each other when they meet. This is an example of the topological charge of BICs^18^; here the two neighboring BICs have opposite charges and can annihilate with each other. Note that near the annihilation of the two BICs, quantitative prediction of the quality factor using FDTD becomes exceedingly hard due to an increased sensitivity on structural variations (which requires an unusually high spatial resolution in FDTD); nonetheless, we can still calculate *Q* efficiently with our method.

## Discussion

In principle, more BICs can lie above the yellow shaded area in Figs 2a and 3a, where there are two or more radiation ports. In this case, three or more independent equations have to be satisfied simultaneously: *f* ′ = 0, *T*_0_ = 0, *T*_1_ =0 … It will require root-finding with more variables than *k*_*y*_ and ω, which means the structure itself has to be fine-tuned to find BICs; such structure-sensitive BICs are less practical as experimental realization will be harder.

Our analysis of PhC slabs with 1D periodicity can be considered an extension of the previous topological vortex work^18^ to the 1D parameter space. As shown in Fig. 4b, here BICs correspond to nodal points where the radiation amplitude crosses zero (instead of vortex centers), which are manifestations of topological charges in 1D.

The preceding examples concern structures where the dielectric is real and symmetric in *y*, for which the matrices **H**_*l*_ are real symmetric and so the function *f* ′ is real-valued. However, BICs can exist in even more general systems. As long as 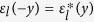, ***H***_*l*_ is real (although not necessarily symmetric and not necessarily Hermitian). In such PT-symmetric systems, if the non-Hermiticity is below the PT-breaking threshold, the eigenvalues and eigenvectors of **H**_*l*_ can still be real, and the function *f* ′ can still be real-valued. Such systems can also support BICs. However, if the introduction of gain leads to lasing, one will need to account for the nonlinearity resulting from gain saturation^37^,^38^,^39^, which is beyond the linear model considered in this work.

## Conclusion

We have presented a mode expansion method that can efficiently and quantitatively describe guided resonances and BICs in PhC slabs, and the method also reveals their underlying formation mechanisms. We find symmetry-protected BICs at the Γ point and at the zone edge, as well as BICs not protected by symmetry. The formalism is easily extendable and applicable to a wide range of structures. This is an attractive approach for the study of guided resonances and BICs in periodic structures.

## Additional Information

**How to cite this article**: Gao, X. *et al.* Formation mechanism of guided resonances and bound states in the continuum in photonic crystal slabs. *Sci. Rep.* **6**, 31908; doi: 10.1038/srep31908 (2016).
